# Evaluating Malaria Prevalence Using Clinical Diagnosis Compared with Microscopy and Rapid Diagnostic Tests in a Tertiary Healthcare Facility in Rivers State, Nigeria

**DOI:** 10.1155/2018/3954717

**Published:** 2018-04-22

**Authors:** M. N. Wogu, F. O. Nduka

**Affiliations:** Department of Animal and Environmental Biology, University of Port Harcourt, Port Harcourt, Nigeria

## Abstract

The World Health Organization's policy on laboratory test of all suspected malaria cases before treatment has not yielded significant effects in several rural areas of Sub-Saharan Africa due to inadequate diagnostic infrastructure, leading to high morbidity and mortality rates. A cross-sectional randomized study was conducted to evaluate the validity of clinical malaria diagnosis through comparison with microscopy and rapid diagnostic test kits (RDTs) using 1000 consenting outpatients of a tertiary hospital in Nigeria. Physicians conducted clinical diagnosis, and blood samples were collected through venous procedure and analyzed for malaria parasites using Giemsa microscopy and RDT kits. Microscopy was considered the diagnostic “gold standard” and all data obtained were statistically analyzed using Chi-square test with a *P* value <0.05 considered significant. Malaria prevalence values of 20.1%, 43.1%, and 29.7% were obtained for clinical diagnosis, microscopy, and RDTs, respectively (*P* < 0.05). Values of 47.2%, 95.9%, and 77.8% were obtained for sensitivity, specificity, and diagnostic accuracy, respectively, in clinical diagnosis, while RDTs had sensitivity, specificity, and diagnostic accuracy values of 73.7%, 97.3%, and 88.3%, respectively, when compared to microscopy (*P* < 0.05). Clinical diagnosed malaria cases should be confirmed with a parasite-based laboratory diagnosis and more qualitative research is needed to explore why clinicians still use clinical diagnosis despite reported cases of its ineffectiveness.

## 1. Introduction

Malaria infection causes high levels of morbidity and mortality in Sub-Saharan Africa especially Nigeria [1]. The World Health Organization (WHO) previously advised presumptive diagnosis as the basis for the first-line treatment of uncomplicated malaria in places where a parasitological test is not possible [1]. This policy allowed uncomplicated malaria illness to be treated by village health workers, shop keepers, and relatives in the home and thus minimizes delays in treatment, especially for those living a long distance from formal healthcare facilities. In most regions in Africa, over 70% of individuals with symptomatic malaria do not initially seek medical assistance from healthcare facilities but are self-diagnosed and receive treatments at home with either traditional medications or antimalarial drugs purchased from local chemists or drug shops [2]. Such symptomatic malaria individuals only seek further treatment in health facilities when self-medications with traditional or orthodox drugs fail and this has a negative effect on the performance of some malaria diagnostic techniques as well as treatment [3]. The signs and symptoms as well as physical examination of the patients by physicians play a key role in clinical diagnosis because clinical diagnosis is still used for therapeutic care of most febrile individuals by physicians in several malaria endemic regions despite some reported cases of its imprecision. Improving the diagnosis of malaria so that therapeutic care is given to only patients who require it is a public health priority in Africa especially Nigeria. Early malaria symptoms vary and are not malaria-specific; they include high fever, headache, general body weakness, recurrent chills, dizziness, abdominal cramps, diarrhoea, nausea bouts, vomiting, and loss of appetite. Other common but equally life threatening infections such as febrile ailments and viral and bacterial infections possess similar signs and symptoms to malaria; hence, clinical diagnosis is quite challenging and unreliable [4]. The signs and symptoms of malaria and other tropical diseases overlap, and this hinders the rate of diagnostic specificity and sensitivity, thereby increasing the wrong use of antimalarial drugs and reduction in the effective management of patients with nonmalarial febrile illness especially in malaria endemic regions [4–6]. Healthcare providers with little diagnostic training in developing countries that also lack adequate laboratory diagnostic equipment have been able to manage and diagnose most childhood diseases due to the clinical algorithms created by the Integrated Management of Children Illness (IMCI) [6]. In an African region, a commonly used clinical algorithm for malaria diagnosis when compared with a fully trained paediatrician with full access to adequate laboratory diagnostic equipment showed a very low specificity rate of 0–9% but a high sensitivity rate of 100% [7, 8]. The low specificity rate shows the diagnostic challenge of differentiating malaria from other types of fever in children based on only presumptive diagnosis. Hence, efficient malaria diagnosis is achieved by using both presumptive and laboratory based (identification of the parasite) diagnosis [9]. The World Health Organization currently recommends that all suspected cases of malaria must undergo laboratory parasite-based diagnosis (e.g., using microscopy and rapid diagnostic test kits) before treatment due to inadequate information on the effectiveness of presumptive diagnosis. This study was conducted to investigate the validity of clinical diagnosis by physicians as an effective malaria diagnostic technique when compared with microscopy and rapid diagnostic test kits in a tertiary hospital in Rivers State, Nigeria.

## 2. Materials and Methods

### 2.1. Study Area and Population

The study was conducted in Port Harcourt, Rivers State. Port Harcourt is the capital of Rivers State, it lies along the Bonny River in the Niger, Delta region of Nigeria, temperature throughout the year is relatively constant (25°C–28°C), relative humidity fluctuates between 90 and 100%, and it is geographically located at latitude 4.75°N and longitude 7.00°E [10]. A total of 1000 consenting study subjects (irrespective of age and sex) attending the Outpatient Department of University of Port Harcourt Teaching Hospital, Port Harcourt, Rivers State, were recruited for the study. Ethical clearance was obtained from the Rivers State Ministry of Health and the University of Port Harcourt Teaching Hospital before the commencement of this study. The inclusion criteria for this study were that study participants had suspected body temperatures ≥ 38°C for less than 10 days, were examined by a physician, and gave their oral or written consent (consent was obtained from the parents or guardians of participants below 18 years) to be part of the research. Exclusion criteria included individuals undergoing malarial treatment (or took antimalarial drugs within two weeks before the research), those diagnosed with mental illness, measles, chickenpox, infected wounds, and pneumonia, and those with suspected body temperatures ≥38°C by physicians but did not give their consent to be part of the study.

### 2.2. Data Collection

Clinical diagnosis was conducted by physicians. Blood samples were collected through venous procedure by trained laboratory scientists and stored in Ethylene Diamine Tetra Acetate (EDTA) tubes to prevent coagulation. From the collected blood samples, blood films (thick and thin) were prepared, stained (using Giemsa stain), and examined microscopically using established laboratory procedures [11]. Thick blood films were used to estimate the level of parasitaemia by counting the number of parasites against 200 white blood cells with the assumption that each subject had 8,000 white blood cells/*μ*L of blood. A minimum of 200 fields were examined before declaring slides negative for malaria parasite. The RDT kit used was CareStart™ malaria HRP2/pLDH Pf Test Kit (Access Bio Inc., USA) and the usage was according to the manufacturer's instructions. For quality assurance when conducting microscopy, two trained microscopists viewed each blood film before declaring the slide positive or negative but when conflicting results arose, a third senior microscopist was used. The RDT kits were stored at the manufacturer's recommended temperature (<40°C) and quality control and the validity of each kit used was certified by several laboratory scientists. Data accuracy was ascertained by double entry of all data obtained.

### 2.3. Data Analysis

The data obtained for clinical diagnosis were compared with those for RDT using microscopy as the gold standard to evaluate sensitivity, specificity, diagnostic accuracy, positive predictive value, and negative predictive value using true positives, true negatives, false positives, and false negatives. Data generated were analyzed using Chi-square test and a *P* value less than 0.05 was considered significant.

## 3. Results

*Plasmodium falciparum* was the only species of malaria parasite identified with confirmation from polymerase chain reaction technique (PCR). Prevalence values of 20.1%, 43.1%, and 29.7% obtained for clinical diagnosis, microscopy, and RDTs, respectively (*P* < 0.05) (Table 1). Using microscopy as the diagnostic standard, clinical diagnosis had value of 47.2%, 95.9%, and 77.8% for sensitivity, specificity, and diagnostic accuracy, respectively, while RDT had sensitivity, specificity, and diagnostic accuracy values of 73.7%, 97.3%, and 88.3%, respectively (*P* < 0.05) (Table 2).

## 4. Discussion

The only malaria parasite observed in this study was* Plasmodium falciparum*. This observation agreed with reports from some similar studies which identified only* P. falciparum* as the only malaria parasite present [12, 13]. According to the World Health Organization,* P. falciparum* is the most prevalent malaria parasite in most regions in Sub-Saharan Africa [14]. The overall malaria prevalence in this study using Giemsa microscopy as the gold standard was 43.1%. The study prevalence is comparable to 46.6% reported in Zamfara State [15], 40.8% in Rivers State [16], and 40.5% in South-Eastern Nigeria [17] but lower than 72.5% in Rivers State [18], 85.7% in Enugu State [19], and 71.4% in Cross River State [20]. The study prevalence (43.1%) is higher than 15.0% in Ogun State [21] and 14.7% in Lagos [22]. The prevalence of malaria in this study was significant and could be attributed to some environmental conditions such as temperature and humidity of the study area;* Anopheles* species thrive well in areas with warm temperature (25°C–28°C) and high relative humidity (90–100%) as well as lack of use of mosquito nets and blocked drainage facilities (which cause flooding and accumulation of stagnant water bodies during intense rainfall, thus increasing breeding and competence levels of* Anopheles* species). The sensitivity, specificity, and diagnostic accuracy recorded for clinical diagnosis was lower than those of RDT using microscopy as the diagnostic standard. There is need to review the use of clinical diagnosis due to its significant underdiagnosis recorded in this study, although some physicians in most malaria endemic regions claim that prompt treatment of suspected malaria cases (especially in children) reduces the progression of mild malaria to severe malaria. The continual usage of clinical diagnosis alone could have detrimental effects like drug abuse and malaria parasite resistance to antimalarials. RDTs were created to address some shortcomings of microscopy, and some previous similar studies reported low sensitivity (below 100 parasites/*μ*L) and diagnostic accuracy for RDTs [23, 24] but data from this study showed that RDTs (if used and stored properly) had a significant higher sensitivity and diagnostic accuracy than clinical diagnosis. However, RDTs should be used to complement microscopy or alone when expert microscopy is unavailable especially in rural malaria, endemic areas of developing countries which lack well-equipped laboratories or expert microscopy for malaria diagnosis. It is vital to properly diagnose malaria especially in malaria-endemic regions, because it will help improve the diagnosis and treatment of other febrile (nonmalaria) infections and limit antimalarial usage to only malaria parasite-based test true positives. The findings from this study buttress the World Health Organization's policy that all clinical diagnosis must be confirmed by a laboratory parasite-based diagnosis before the administration of antimalarials to prevent malaria misdiagnosis and drug resistance.

## 5. Conclusion

Clinical diagnosis is not a reliable malaria diagnostic technique especially in Sub-Saharan Africa due to inadequate local epidemiological data on malaria and the presence of other febrile ailments which possess similar signs or symptoms with malaria. Therefore, all suspected malaria cases (clinical diagnosis) should be confirmed with a laboratory test.

## Figures and Tables

**Table 1:** Malaria prevalence in relation to diagnostic techniques.

Diagnostic techniques	Number examined	Number infected (%)
Clinical diagnosis	1000	201 (20.1)
Microscopy	1000	431 (43.1)
RDTs	1000	297 (29.7)

*X* ^2^ = 124.855, Df = 2, and *P* value = 0.001 (*P* < 0.05).

**Table 2:** Diagnostic efficiency of presumptive diagnosis and RDTs using microscopy as a standard.

Diagnostic techniques	Diagnostic parameters (%)				
	SN	SP	DA	PPV	NPV
Clinical diagnosis	47.2	95.9	77.8	87.1	75.5
Microscopy	100	100	100	100	100
RDTs	73.7	97.3	88.3	94.3	85.8

SN = sensitivity; SP = specificity; DA = diagnostic accuracy; PPV = positive predictive value; NPV = negative predictive value; and *X*^2^ = 10.150, Df = 4, and *P* value = 0.038 (*P* < 0.05).
